# A case of radiation treatment effect mimicking viable, recurrent meningioma on DOTATATE PET imaging

**DOI:** 10.1093/noajnl/vdaf244

**Published:** 2025-11-20

**Authors:** Brooke C Braman, Kanish Mirchia, Javier E Villanueva-Meyer, David R Raleigh

**Affiliations:** Department of Radiation Oncology, University of California San Francisco, San Francisco (B.B., D.R.); Department of Pathology, University of California San Francisco, San Francisco (K.M., D.R.); Department of Neurological Surgery, University of California San Francisco, San Francisco (J.V., D.R.); Department of Radiology, University of California San Francisco, San Francisco (J.V.); Department of Radiation Oncology, University of California San Francisco, San Francisco (B.B., D.R.); Department of Pathology, University of California San Francisco, San Francisco (K.M., D.R.); Department of Neurological Surgery, University of California San Francisco, San Francisco (J.V., D.R.)

**Keywords:** DOTATATE, imaging, Meningioma | MRI, PET, radiation necrosis

**SSTR2-directed positron emission tomography (PET) with radiotracers such as DOTATATE has emerged as a sensitive molecular imaging modality for meningioma, but the specificity of SSTR2-directed PET for meningioma is incompletely understood. Here we report a case of DOTATATE PET uptake in pathology-proven brain parenchyma with radiation treatment effect. DOTATATE PET and magnetic resonance imaging (MRI) were used for surveillance in this patient with a history of recurrent meningioma status post multiple prior resections and two courses of radiotherapy. DOTATATE PET identified multiple areas of extra-axial avidity concerning for meningioma recurrence, as well as an avid lesion in the temporal lobe brain parenchyma that was located immediately adjacent to the prior resection cavity and radiotherapy fields. At the time of re-resection, the temporal lobe lesion demonstrated only brain parenchyma with radiation treatment effect, though other DOTATATE PET-avid areas were pathology-proven as recurrent meningioma. To our knowledge, this is the first report of radiation treatment effect mimicking viable, recurrent meningioma on DOTATATE PET. Thus, this case report describes an under-recognized non-neoplastic process with DOTATATE PET avidity that can yield false positive results, highlighting the essential role of multimodality imaging for optimal meningioma surveillance.** 

Meningioma is the most common primary brain tumor.^1^ Surgery is the backbone of meningioma treatment, with radiotherapy reserved for cases with subtotal resection, high-risk disease, or at the time of recurrence.^2^ Post-treatment surveillance imaging is essential as rates of local recurrence are 50% or higher for central nervous system World Health Organization (CNS WHO) grade 2-3 meningiomas.^3^ Anatomic imaging with contrast-enhanced magnetic resonance imaging (MRI) is the gold standard for meningioma detection and post-treatment surveillance, but molecular imaging with DOTATATE, DOTANOC, or DOTATOC positron emission tomography (PET) offers high sensitivity and is increasingly used in clinical practice.^2^^,^^4^^,^^5^ In the United States, [^68^Ga]-DOTATE and [^64^Cu]-DOTATATE are clinically available,^6^ and [^64^Cu]-DOTATATE has been more recently developed and is increasing in use due its longer half-life and reported greater sensitivity for lesion detection.^7^^,^ ^8^ These imaging agents use radiolabeled somatostatin receptor (SSTR) analogs that bind SSTR2, which is the most sensitive and specific immunohistochemical marker for meningioma.^9^ Other neoplastic and non-neoplastic tissues can express SSTRs, including neuroendocrine tumors, pituitary adenomas, medulloblastoma, normal brain parenchyma, and inflammatory cells, and may also appear avid by SSTR2-directed PET, potentially impacting imaging interpretation.^10-12^ Here we report a case of DOTATATE PET avidity in a region of pathology-proven brain parenchyma with radiation treatment effect in a patient with multiple meningioma recurrences.

A 76-year-old female underwent gross total resection of a right sphenoid wing meningioma, CNS WHO grade 2, showing 7 mitoses per 10 high-power fields (HPF), Ki-67 labeling index of 20%, and focal brain parenchymal invasion. Eight months later, surveillance MRI identified recurrence at the anterior resection cavity that was treated with stereotactic radiosurgery to a total dose of 25 Gray (Gy) in 5 fractions (Fx). Further progression at the anterior resection cavity was noted 2 years later, and she underwent near-total re-resection of recurrent CNS WHO grade 2 meningioma infiltrating the skull base with up to 9 mitoses per 10 HPF and Ki-67 labeling index of 15%. Next-generation DNA sequencing (NGS) showed *NF2* mutation and losses of chromosomes 1p, 6q, and 22q, and gains of chromosomes 1q and 17q. Adjuvant radiotherapy was used to treat the resection cavity to 54 Gy in 30 Fx with a simultaneous integrated boost to 63.6 Gy to residual disease.

Twenty months later, [^64^Cu]-DOTATATE PET/MRI imaging (4.02 mCi with 85-minute uptake time) was concerning for further progression along the right skull base (SUV_max_ 31.7, SUVR_SSS_ 35.6) and a separate focus of DOTATATE avidity in the medial right temporal lobe (SUV_max_ 4.6, SUVR_SSS_ 5.2) that was also felt to represent recurrent disease (Figure 1A). Concurrently acquired contrast-enhanced MRI showed increased enhancement along the right sphenoid wing, favored to represent recurrent disease, and new necrotic parenchymal enhancement involving the medial right temporal lobe with surrounding edema, favored to represent radiation necrosis (Figure 1A). Extensive subtotal re-resection was performed, and intra-operatively, the temporal lobe appeared gliotic, and abnormal bone and dura were encountered in the middle cranial fossa. Specimens from the skull base demonstrated recurrent meningioma, CNS WHO grade 2, with rare mitoses and Ki-67 labeling index of 8% (Figure 1B). Repeat NGS showed a similar copy number profile compared to prior, but with new homozygous/­biallelic *CDKN2A/B* deletion, thereby meeting criteria for ­meningioma, CNS WHO grade 3. The temporal lobe ­resection specimen did not show any involvement by meningioma and was entirely composed of brain parenchyma with vacuolization and reactive astrogliosis, necrosis, scattered foamy macrophages, hyalinized blood vessels, and rare, atypical cells, compatible with radiation necrosis (Figure 1C).

**Figure 1.**
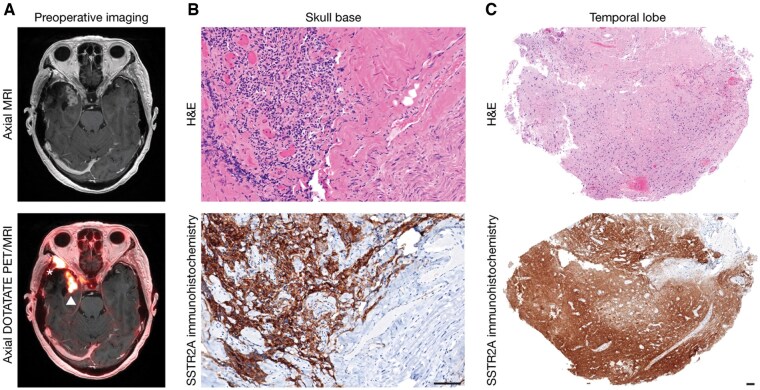
**A case of radiation treatment effect mimicking viable, recurrent meningioma on DOTATATE PET imaging. (A**) Preoperative axial post-contrast MRI (top) and DOTATATE PET/MRI (bottom) show DOTATATE PET-avid, MRI-enhancing lesions in the right sphenoid wing (*) and medial right temporal lobe (arrowhead). (**B**) Hematoxylin and eosin (H&E)-stained tissues from the skull base (top) with corresponding SSTR2A immunohistochemistry (UMB1 clone [ab134152, Abcam, 1:2000 dilution]) (bottom) highlight meningioma cells. Scale bar, 100 µm. (**C**) H&E-stained tissues from the temporal lobe (top) with corresponding SSTR2A immunohistochemistry (bottom). Scale bar, 100 µm.

In this patient with multiple meningioma recurrences, DOTATATE PET uptake reflected radiation treatment effect instead of viable meningioma in a parenchymal lesion that co-occurred with nearby extra-axial meningioma. While the mechanism underlying SSTR2 expression in radiation necrosis remains unclear, non-neoplastic DOTATATE PET avidity has been reported in inflammatory processes, likely owing to SSTR expression on lymphocytes and macrophages.^10^^,^^12^ Enrichment in inflammatory cells with SSTR expression was considered as a possible explanation here. However, lymphocytes and macrophages were not a significant component of the examined tissue. SSTR2A immunohistochemistry was performed on multiple specimens from this patient. Specimens involved by meningioma showed strong positivity in meningioma cells as well as extensive cellular and parenchymal staining in the non-neoplastic brain (Figure 1B). The temporal lobe specimen showed extensive granular staining in neutrophils with no significant increase in foamy macrophages (Figure 1C).^13^ Thus, inflammatory infiltrate did not explain non-meningioma DOTATATE PET avidity here, but this remains a possibility in other cases. Inflammation should be considered when interpreting DOTATATE PET, especially if performed post-operatively or in the weeks to months after radiotherapy when active local inflammation is expected.

SUV interpretation is a key component in distinguishing meningioma and non-meningioma DOTATATE PET avidity. Current consensus recommendations establish thresholds of SUV <2.3 or SUVR_SSS_ <3 for differentiating meningioma from non-tumor tissue using [^68^Ga]-DOTATATE PET. These thresholds are based on limited sample sizes, and no published thresholds exist for [^64^Cu]-DOTATATE in meningioma.^6^

This is the first report of radiation treatment effect mimicking viable, recurrent meningioma on DOTATATE PET, where contrast-enhanced MRI was discordant and offered superior diagnostic discrimination of the underlying pathology. Given the greater sensitivity and increasing clinical adoption of [^64^Cu]-DOTATATE, this case also underscores the urgent need for further investigation of semi-quantitative assessment parameters and diagnostic thresholds specific to this radiotracer to improve diagnostic accuracy in post-treatment surveillance. This case highlights a potential diagnostic pitfall when using DOTATATE PET after radiotherapy for meningioma and reinforces the importance of using both molecular and anatomic imaging for meningioma surveillance. The incompletely defined specificity of DOTATATE PET in meningioma may be relevant to emerging theranostics. Lutetium-177-DOTATATE (^177^Lu-DOTATATE), a DOTATATE-bound radionucleotide, has shown promise in refractory meningioma in a phase II trial.^14^ Off-target effects of SSTR targeted therapies will require close monitoring as they are investigated in meningioma.

## Data Availability

Not applicable.
